# Exploration and Comparison of the Behavior of Some Indigenous and International Varieties (*Vitis vinifera* L.) Grown in Climatic Conditions of Herzegovina: The Influence of Variety and Vintage on Physico-Chemical Characteristics of Grapes

**DOI:** 10.3390/plants12040695

**Published:** 2023-02-04

**Authors:** Tatjana Jovanović-Cvetković, Milica Sredojević, Maja Natić, Rada Grbić, Milica Fotirić Akšić, Sezai Ercisli, Miljan Cvetković

**Affiliations:** 1 Faculty of Agriculture, University of Banja Luka, 78000 Banja Luka, Bosnia and Herzegovina; 2 Innovative Centre Faculty of Chemistry Belgrade, University of Belgrade, 11158 Belgrade, Serbia; 3 Faculty of Chemistry, University of Belgrade, 11000 Belgrade, Serbia; 4 Department of Fruit Science and Viticulture, Faculty of Agriculture, University of Belgrade, 11080 Belgrade, Serbia; 5 Department of Horticulture, Faculty of Agriculture, Ataturk University, Erzurum 25240, Turkey

**Keywords:** climate, bunch, grape berry, quality, phenolics

## Abstract

Viticulture is of great economic importance in the southern part of Bosnia and Herzegovina, thanks to favorable climatic conditions and a long-standing tradition of growing vines. The assortment is dominated by international varieties, as well as some autochthonous and domesticated varieties. The subject of the research is the analysis of the quality of Cabernet Sauvignon, Merlot, Vranac, and Blatina varieties at two localities in Herzegovina during the period 2020–2021. The paper examined the most important economic and technological characteristics, grape quality, and berry phenolic profile. A particularly pronounced variation of the tested characteristics during the research period was observed in the Merlot and Blatina varieties, while the Cabernet Sauvignon and Vranac varieties showed a slightly higher stability of the tested characteristics. Poorer grape quality during the research period was registered with the Blatina variety, which can be considered a varietal characteristic to some extent. The analyzed grape varieties were rich in polyphenols, and the impact of grape variety on the berry phenolic profiles was confirmed. The most abundant polyphenols in the analyzed grape samples were quercetin 3-*O*-glucoside and catechin gallate, followed by kaempferol 3-*O*-glucoside. The highest values of polyphenols were found mainly in the samples originating from Trebinje. Indigenous Balkan grape varieties (Vranac and Blatina) stood out with particularly high contents of some phenolics. Research has shown that climatic conditions have a significant influence on the most important characteristics of grapes, which are conditioned by genotypic specificities. The conditions for growing vines in the conditions of Herzegovina enable high quality in the production of grapes, especially the Cabernet Sauvignon and Vranac varieties. The autochthonous variety Blatina shows significant variations in grape quality during the test period, which was confirmed by the results of a larger number of studies in the previous period.

## 1. Introduction

The grape (*Vitis vinifera* L.) and wine production in Bosnia and Herzegovina (B&H) is primarily linked to the region of Herzegovina. Favorable natural conditions for grapevine cultivation, as well as the traditional orientation of the local population towards viticulture and wine production, created a centuries-old tradition of grape and wine production [1]. The basic prerequisite for successful grape production is the use of an adequate variety with good genetic potential for obtaining optimal yield and quality. Choosing the right grape variety is of great importance in modern viticulture.

Each grapevine variety has a unique combination of characteristics, which together with applied agrotechnical and ampelotechnical measures, geographical locality, and ecological characteristics of the locality, as well as vinification techniques, affect the quality of grape and produced wine [2]. In the wine-growing region of Herzegovina today, various varieties of grapevine are grown. On the basis of [3], a significant number of autochthonous grapevine varieties have been recorded in the territory of Herzegovina, among which there are a smaller number of commercial and significantly more non-commercial, mostly old, and less cultivated varieties. Next to the Žilavka, the Blatina variety has the greatest oenological and economic importance [4].

Knowing the grape cluster and grape berry characteristics is important for evaluating not only the ampelographic but also the technological characteristics of the grape varieties. The structural and physico-chemical characteristics of the grape cluster, that is, the grape berry, are among the factors that determine the grape and wine quality. Grape quality is associated with biological variability that occurs at multiple levels: between vineyards, grapevines of the same vineyard, grape clusters of the same grapevine, and even among grape berries of the same grape cluster [5]. The size of grape cluster and grape berries is primarily determined by the development and growth process during the vegetation period. A large number of studies analyze the influence of various factors (genetic, agroecological, agrotechnical) on the size, number, and structure of grape clusters and berries, as well as the further influence of these parameters on the grape and wine quality [6,7,8,9,10,11]. According to [12], the number of berries in a cluster is the product of the number of flowers in an inflorescence, i.e., the percentage of flowers that produce fruit and, together with the grape berries weight, it is greatly influenced by the temperature and rainfall during the flowering period. There are great differences in the level of seed development in different varieties of *Vitis vinifera* L., where in parthenocarpy varieties seed development does not occur at all, unlike other varieties in which complete seed development occurs from a few ovules [13]. Many studies show that there is a positive linear relationship between the total seed weight, i.e., the number of seeds and the grape berry weight [14,15]. During the stages of berry growth, along with the processes that determine physical properties, biochemical activities take place, namely, the accumulation of primary (sugars and acids) and secondary metabolites (phenolic compounds). Their composition, quantity, and mutual relationship consequently affect the grape quality. The occurrence of anthocyanins in the berry skin (véraison) marks the beginning of the ripening period, when there is a drop in the concentration of certain compounds formed in the first phase (malic acid, tannin) and a large jump in the concentration of sugars, glucose, and fructose, which are products of the enzymatic hydrolysis of sucrose transported from the leaves into berries [16,17]. According to [18], “harvest quality” depends mainly on the content of sugar and acids in grape and their composition in colored and aromatic compounds, while the quantitative expression of these qualitative elements mainly depends on the interaction of climate and genotype, so that we rarely encounter similar results in terms of quality, in different years and different localities. According to the results of some research, the content and composition of sugars and acids in grapes was primarily conditioned by the genotype [19,20], and the influence of climatic conditions in the years of research was more reflected in the acids. Similar results were obtained earlier by [21], where an increase in temperature from 15 to 30 °C did not lead to a significant increase in sugar concentration, in contrast to titratable acidity, whose level decreased under the given conditions.

Grape berries represent a rich source of polyphenols that are classified as flavonoids and non-flavonoids [22]. The application of advanced MS technologies in the research of grapes and wines improved knowledge of grape polyphenols. These functional bioactive compounds contribute to nutritional value, organoleptic properties, antioxidant properties, and responses to biotic and abiotic stresses in grapes [23].

Anthocyanins are present only in red grape varieties and they are responsible for skin color [24]. They are important indicators for chemotaxonomic studies and for evaluating the stage of grape ripening. Flavonols are found in all *Vitis* species, and they possess properties of photoprotection and co-pigmentation (with anthocyanins).

The presence of this class of flavonoids is often an indicator of the grape quality. Flavan-3-ols are mostly located in grape seeds, followed by the skins, while phenolic acids are the most abundant in pulp [25]. Phenolic composition is influenced by differences in grape varieties, environmental conditions, and cultural practices [23]. The agroecological factors that most influence the phenolic profiles of the grapes are geographic origin, climate, soil compositions, exposure to diseases, and degree of ripeness. Polyphenols are proven to offer a series of health benefits for humans, to prevent degenerative diseases, or to assist traditional treatments [26]. These grape phytochemicals possess anti-inflammatory, cardio-protective, neuro-protective, anti-proliferative, anti-cancer, and metabolic syndrome protective effects [27].

The present study aimed to determine grape cluster characteristics, grape quality, and phenolic profiles of two international and two indigenous Balkan grape varieties belonging to two different vine-growing areas.

## 2. Results

### 2.1. Climatic Characteristics

**Figure 1:**
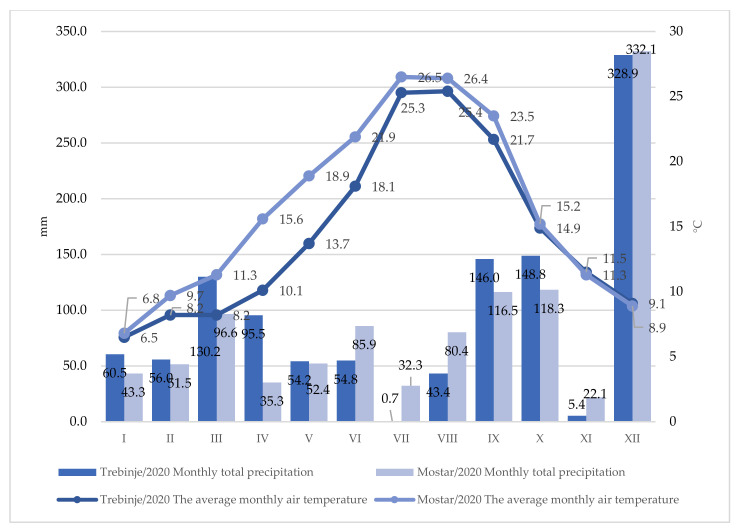
Climatic conditions (total rainfall and average air temperature) at Trebinje and Mostar localities during 2020.

**Figure 2:**
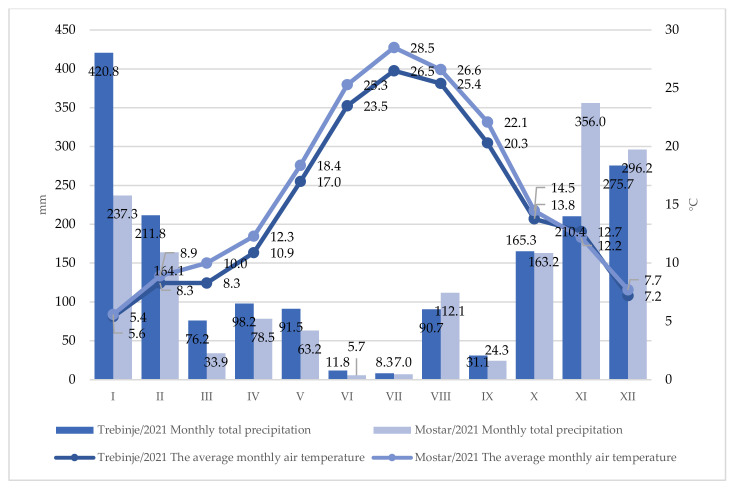
Climatic conditions (total rainfall and average air temperature) at Trebinje and Mostar localities during 2021.

Figure 1 and Figure 2 show the average air temperature for the climatic period of the research by month, for each analyzed year, for the Trebinje and Mostar localities. Basic climate parameters at the locality indicate significant differences between the years of research and the localities themselves. During 2020, slightly higher average air temperatures were registered at the Mostar locality throughout the year, which was especially pronounced at the beginning of the vegetation period (March–May). The amounts of precipitation were relatively uniform, although they were somewhat higher at Mostar locality, during the June–August period.

Moreover, higher amounts of precipitation (in comparison with the summer period) were recorded in September during the period of grape ripening at both localities, and the amount was slightly higher in Trebinje when compared to Mostar. During the year 2021, as expected, the air temperature was somewhat higher at Mostar locality, with a more pronounced difference in the period from June to August (Figure 2). When it comes to precipitation, it was somewhat more uniform, especially during the vegetation period. Precipitation was recorded in most of the vegetation period, except in June and July. A slightly more significant amount of precipitation was recorded during August, while September had less rainfall, which could certainly have a positive effect on the grape quality. Less rainfall during grape ripening has a positive effect on the accumulation of sugar and the formation of better-quality raw material for wine production.

July and August are the warmest months in both localities. During the research period, the warmest month in Mostar was July with an average monthly temperature of 27.5 °C, and in Trebinje, it was July with 25.9 °C, which indicates that the warmest month in Mostar was 1.6 °C warmer than in Trebinje. The coldest month was January with an average monthly temperature of 5.95 °C in Trebinje, and 6.2 °C at the Mostar locality. For a better assessment of climate parameters during the research period, an assessment of temperature (annual average, average maximum, and average minimum), precipitation, and growing degree days for the research period was performed in comparison with the multi-year average (2010–2019) for Trebinje (Table 1) and Mostar (Table 2) localities.

Average annual air temperature for Trebinje during the climatological period 2000–2019 (marked as 00/19 in the table) was 15.7 °C, and for Mostar was 16.1 °C, which indicates that the average annual temperature for Mostar was higher by 0.4 °C compared to the Trebinje locality. In the same period, the average temperature for the vegetation period for Trebinje was 20.8 °C, and for Mostar was 21.4 °C, which indicates that the average vegetation temperature in Mostar compared to Trebinje was higher by 0.6 °C. In the research years (2020 and 2021), the mean annual temperature for Trebinje was 14.4 and 14.9 °C, and the mean vegetation temperature was 20.0 and 19.6 °C. Comparing with the values for the period 2000–2019, it can be concluded that in the years of the study, the mean annual temperature was lower, especially in 2020 by 1.31 °C.

For the locality of Mostar, the average annual temperatures for the research period (2020–2021) were 16.3 and 16.0 °C, which were quite similar to the average annual temperature for the period 2000–2019 (16.1 °C). The mean vegetation period temperature for Mostar locality in 2020 and 2021 was identical (21.1 °C), so it can be concluded that there was no fluctuation in the mean vegetation period temperature at this locality, both in the years of research and, also, compared to the period 2000–2019 (21.4 °C).

Average maximum vegetation temperatures during the research period were 26.1 °C (2020) and 26.0 °C (2021) for Trebinje locality, and 27.4 °C (2020) and 27.8 °C (2021) for Mostar locality. The average maximum vegetation period air temperatures at the Mostar locality were higher in the years of the research compared to the stated temperatures for the Trebinje locality (1.4 °C in 2020, and 1.8 °C in 2021). In comparison with the values for the period 2000–2019, it can be concluded that the year 2020 was normal for both localities, while in 2021, the mean maximum vegetation temperature in Mostar was higher for 0.4 °C compared to the period 2000–2019.

The climate of the wine-growing localities in Trebinje and Mostar is classified in region V, with 2372.7 °C (Trebinje) and 2555.3 °C (Mostar) (climate period 2000–2019). This region covers the interval of 2220.0 °C to 2700.0 °C. In the years of research (GDD) for the Trebinje locality was 2140 °C in 2020 and 2054.4 °C in 2021. The GDD for the Mostar locality was higher than the Trebinje locality, and it value was 2375.4 °C for both years. The annual amount of precipitation for the climatological period 2000–2019 was 1683.7 mm in Trebinje and 1491.6 mm in Mostar. During the vegetation period, the amount of precipitation in the period April–October for the Trebinje locality was 705.3 mm, and for the Mostar locality was 685.8 mm. In 2020, 1124.4 mm of precipitation was recorded at the Trebinje locality, 543.4 mm during the vegetation period, while in 2021, the annual amount of precipitation was 1691.8 mm, i.e., 496.9 mm during the vegetation period. At the Mostar locality in 2020, 1066.7 mm was recorded during the year, 521.1 mm during the vegetation period, while in 2021, the total amount of precipitation was 1541.5 mm, 454.0 mm during the vegetation period. The comparative analysis of the multi-year average and the years of research indicates that in the research years, we had a lower amount of precipitation in the vegetation period, as well as in the months of grape ripening (August–September), and it should be noted that the year 2020 had a higher amount of precipitation, both in the vegetation period as well as in the months of grape ripening, compared to 2021.

### 2.2. Grape Cluster Characteristics

The highest grape cluster weight during the research period at the Trebinje locality (Table 3) was recorded in the Blatina variety in 2020 (373.9 g), while the lowest grape cluster weight was recorded in the Merlot variety in 2021 (173.9 g). The average cluster weight of the analyzed varieties was statistically significantly influenced by the research year and the analyzed varieties.

The analyzed varieties had a slightly higher cluster weight during 2020. The Blatina and Vranac varieties had the highest cluster weight during the research period, and there was no statistically significant difference between them. The cluster weight of these varieties was statistically significantly higher compared to the Cabernet Sauvignon and Merlot varieties.

The number of gape berries in a cluster is primarily a varietal specificity, which is significantly influenced by the year of research, as well as their interaction (Table 3). In 2020, the Vranac variety numerically had the lowest number of grape berries in a grape cluster (139.0), which was less compared to the other varieties, and this difference was statistically significantly smaller compared to the Cabernet Sauvignon and Merlot varieties. During 2021, the number of berries in a cluster was slightly lower and more uniform in the observed varieties and ranged from 115.2 (Blatina) to 155.2 (Cabernet Sauvignon).

The weight of ten grape berries was strongly influenced by variety, research year, and their interaction (Table 3). The weight of ten grape berries was statistically significantly higher in 2021 compared to 2020. It was identical and the highest in the varieties Vranac in 2020 and Blatina in 2021 (29.8 g). The Vranac variety had a high weight of ten grape berries also in 2021 (26.6 g). The variety Cabernet Sauvignon had the lowest weight of ten grape berries during the research period (11.4 g in 2020 and 12.8 g in 2021, respectively). The Merlot variety had a relatively uniform weight of ten grape berries during the research period (15.2 g in 2020 and 15.1 g in 2021 respectively). The number of seeds in ten grape berries (Table 1) was not influenced by the research year, unlike the influence that had variety, as well as the interaction of year and variety. The highest number of seeds and ten grape berries was recorded in the varieties Blatina in 2021 (22.8 g) and Vranac in 2020 (22.7 g). The lowest number of seeds in ten grape berries during both years of research was recorded in the Cabernet Sauvignon variety (16.5 g in 2020 and 17.1 g in 2021).

The highest weight of grape cluster during the research period, at the Mostar locality (Table 4), had the Blatina variety in 2021 (428.7), while the lowest cluster weight was observed in the Cabernet Sauvignon variety during both years of the research (152.9 in 2020 and 149.5 g in 2021). The weight of the Vranac variety during both years of the research, as well as the Blatina variety during 2021, were statistically significantly higher than all other analyzed varieties. The year of the research and the specificity of the analyzed varieties, as well as their interactions, had a strong influence on the grape cluster weight.

The analyzed varieties had a higher grape cluster weight during 2021, while the highest average grape cluster weight during the research period was recorded in the Vranac variety, which was statistically significantly higher than the other analyzed varieties. It is particularly interesting to point out the lower grape cluster weight of the Blatina variety in 2020 (229.1 g).

The number of grape berries in a grape cluster at both localities was influenced by the research year and their interaction (Table 4). Most of the analyzed varieties had a slightly higher number of grape berries in the cluster during 2021. The Merlot variety had the highest number of grape berries in a grape cluster during both research years (195.1 in 2020 and 184.4 in 2021), although this difference had statistical significance only in terms of number of grape berries in a grape cluster in the Blatina variety in 2020 (78.9).

The weight of ten berries was strongly influenced by the variety (Table 4), while no statistically significant difference was found in the research years (22.6 g in 2021 and 23.3 g in 2020, respectively). The Blatina variety had the highest number of grape berries in a grape cluster during both research years (33.0 in 2021 and 32.1 in 2020). The Cabernet Sauvignon variety had a uniform and at the same time the lowest number of grape berries in the grape cluster (12.6) during both years of research.

On the number of seeds in ten grape berries (Table 4), the variety itself had the greatest influence and the interaction of the research year and the variety. The highest number of seeds in ten berries was recorded in the Blatina variety in 2020 (25.7 g), while the lowest number of seeds was observed in the Cabernet Sauvignon variety during both research years (15.5 g in 2020 and 16.0 g in 2021).

### 2.3. Grape Quality

The numerically highest total soluble solids (TSS) content during the research period at Trebinje locality (Table 5) was recorded in the Merlot variety (24.8 °Brix in 2020 and 23.1 °Brix in 2022). The lowest TSS content was observed in the Blatina variety (23.4 °Brix in 2020 and 21.5 °Brix in 2021). The variety Vranac also had a slightly lower TSS content during both research years.

The research year had a statistically highly significant effect on the analyzed characteristic, bearing in mind that all analyzed varieties had a higher TSS content during 2020. The varieties Merlot and Cabernet Sauvignon had a higher TSS content compared to the autochthonous varieties Blatina and Vranac. The interaction of genotype and research year had no significant effect on the analyzed characteristic. The titratable acid (TA) content was strongly influenced by the variety and research year, as well as their interaction (Table 5). All analyzed varieties had a slightly higher TA content during 2021. During 2020, the highest TA content was recorded in the Merlot variety (6.1 g/L), while the lowest was observed in the Vranac variety (4.1 g/L). During 2021, the Blatina variety had the highest TA content (7.1 g/L), and the Vranac variety had the lowest (5.6 g/L).

The varieties showed similar behavior when it comes to the pH value (Table 5). The research year, variety, and their interaction had a statistically very significant influence on the pH value. Observed at the year level, the average values were slightly higher in 2021, although the Vranac variety had a uniform value in both years of research, and the Blatina variety recorded a slightly lower value in 2021. The highest pH was recorded in the Blatina variety (3.0 in 2020), which also had the lowest pH value in 2021 (2.9).

The Merlot variety also had the highest TSS content at the Mostar locality (Table 6) during the research (26.3 °Brix in 2020 and 25.9 °Brix in 2021). The Cabernet Sauvignon variety had an almost uniform sugar content during the research period, while pronounced oscillations during the research period were found in the TSS content of the Vranac and Blatina varieties. The Blatina variety had the statistically lowest TSS content (19.1 °Brix in 2020 and 16.6 °Brix in 2021). Apart from the variety, the year of the research also had a statistically significant influence on the TSS content, bearing in mind that this parameter had higher values during 2021. The interaction of variety and research year was statistically highly significant.

The TA content (Table 6) during the research period was the highest in the Blatina variety (6.4 g/L in 2020 and 8.2 g/L in 2021). The Merlot variety had a low TA content during the research period, although it was slightly higher in 2021.

The Cabernet Sauvignon variety had a uniform TA content during the research period, while the Vranac variety showed significant deviations between the research years. Genotype and conditions in the research years had a statistically highly significant influence on the TA content in grapes.

The variety, research year, and the realized interaction had a statistically highly significant influence on the pH value (Table 6). The highest pH values were recorded in the Cabernet Sauvignon variety (3.65 in 2020 and 3.70 in 2021 respectively), while the lowest values were observed in the Blatina variety (2.91 in 2020 and 3.27 in 2021).

### 2.4. Phenolic Profiles of the Grape Samples, TPC, RSA, and TAC

A total of 18 phenolic compounds were identified and quantified in the analyzed grape samples (Table 7). UV chromatograms of grape samples are presented in Figure S1. Quercetin 3-*O*-glucoside and catechin gallate were the most abundant polyphenols in samples, followed by kaempferol 3-*O*-glucoside, rutin, and isorhamnetin 3-*O*-rutinoside.

Among hydroxycinnamic acids, chlorogenic and neo-chlorogenic acids had a uniform content in all samples (ranges of 0.75–2.91 mg/kg and 0.95–2.59 mg/kg, respectively), while the contents of caffeic acid were significantly lower (range from 0.06 to 0.20 mg/kg). Moreover, caffeic acid was the only compound that was not found in one sample (Cabernet Sauvignon, Trebinje 2021). All the other polyphenols were detected in all the analyzed samples. Among 11 detected flavonols, quercetin 3-*O*-glucoside had the highest content in most of the samples. Exceptions were observed in the samples Vranac, Trebinje (both years) and Merlot, Mostar 2021, where isorhamnetin 3-*O*-rutinoside was the most abundant flavonol, and in Vranac, Mostar 2021 (myricetin was the dominant flavonol). Most samples were also rich in rutin, quercetin 3-*O*-rhamnoside, and kaempferol 3-*O*-glucoside (average contents were 6.23 mg/kg, 2.06 mg/kg, and 7.10 mg/kg, respectively). Quercetin, kaempferol, and their dihydrate forms, together with isorhamnetin, were the least represented flavonols in the samples, with contents generally less than 1 mg/kg. Contents of quercetin and kaempferol were notably higher in the two Merlot grape extracts, originating from Trebinje 2021 (2.89 and 1.79 mg/kg, respectively) and from Mostar 2020 (1.52 and 1.28 mg/kg, respectively), when compared to the rest of the samples.

Flavanons (naringenin and eriodictyol) and dihydrochalcone (phlorizin) were found in low concentrations in all samples (range from 0.04 to 0.16 mg/kg). As can be observed from Table 7, the lowest values of TPC and RSA were obtained in the sample Blatina, Mostar 2021 (3.17 g GAE/kg and 25.58 mmol TE/kg, respectively), while the highest results were obtained in Cabernet Sauvignon, Trebinje 2021 (6.76 g GAE/kg and 48.55 mmol TE/kg, respectively). Blatina samples from Trebinje had higher TPC and RSA in comparison to the same variety from Mostar. Vranac and Merlot samples from 2021 had higher TPC and RSA than samples collected in 2020. Vranac samples stood out with the highest TAC values, similar for both localities and years, with the average value of 2.42 g of malvidin-3-glucoside (mal 3-glu)/kg. In the remaining samples, TAC was in the range from 0.67 g mal 3-glu/kg (Blatina, Mostar 2020) to 1.90 g mal 3-glu/kg (Blatina, Trebinje 2020). Blatina samples from Trebinje had a higher content of total anthocyanins when compared to the same grape variety originated from Mostar. On the other hand, Cabernet Sauvignon from Mostar had higher TAC in comparison to the samples from Trebinje. Comparing samples from different harvest years, we found that TAC was higher in Blatina and Cabernet Sauvignon samples from 2020, as well as in Vranac and Merlot grapes from 2021.

### 2.5. Principal Component Analysis (PCA) and Correlation Analysis

Firstly, PCA was applied on the results obtained for total phenolic content, radical scavenging activity, and the content of individual phenols. A dataset consisting of 16 objects (the number of grape samples) × 21 variables (individual polyphenols, TPC, and RSA) was processed using the covariance matrix with auto-scaling (Table S1).

**Figure 3:**
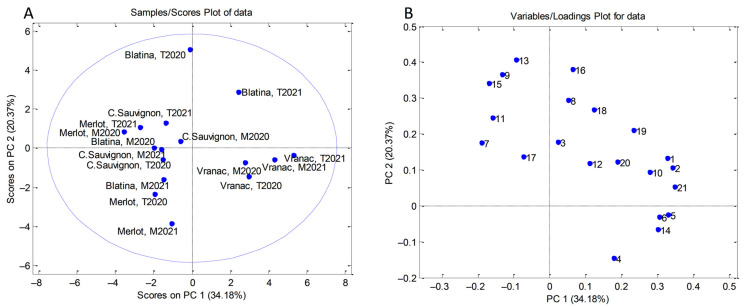
PCA analysis performed on data obtained from phenolic profiles (individual polyphenols, TPC, and RSA) of grape samples: (**A**) score plot and (**B**) loading plot (number of compounds corresponds to Table 7).

PCA resulted in a five-component model that explained 83.35% of total variance. The first principal component accounted for 34.18% of the overall data variance, while for the second and the third, they were 20.37% and 12.29%, respectively. Although the PCA correlation plot (Figure 3A) indicates that a clear separation of the samples based on the phenolic profiles was not achieved, some conclusions can be drawn. All grape samples belonging to the Vranac variety differed from other varieties on the basis of higher contents of neo-chlorogenic acid, rutin, isorhamnetin 3-*O*-rutinoside, myricetin, chlorogenic acid, and TAC (Figure 3B). Blatina grape samples from Trebinje stood out with higher contents of dihydroquercetin, kaempferol 3-*O*-glucoside, isorhamnetin, and naringenin (Trebinje, 2020), as well as phlorizin and quercetin 3-*O*-rhamnoside (Trebinje, 2021).

Additionally, PCA was applied on all experimental data (TPC, RSA, TAC, individual polyphenols, and physicochemical properties of grapes), and the number of variables increased to 44 (16 objects × 44 variables). This PCA resulted in a six-component model that explained 82.17% of the total variance. The first principal component (PC1) accounted for 30.56%, the second (PC2) for 14.67%, and the third (PC3) for 13.18% of the total data variance.

**Figure 4:**
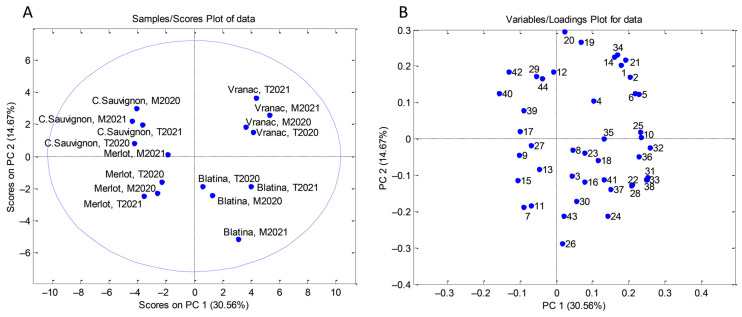
PCA analysis performed on data obtained from phenolic profiles (individual polyphenols, TPC, and RSA) and physicochemical parameters of grape samples: (**A**) score plot and (**B**) loading plot (number of compounds corresponds to Table 7 and Table S1).

When PCA was applied to all parameters (Figure 4A), Vranac and Blatina were separated along the PC1 axis from Cabernet Sauvignon and Merlot (Figure 4B) due to higher contents of quercetin 3-*O*-rhamnoside, chlorogenic acid, neo-chlorogenic acid, and some physicochemical parameters (grape cluster weight, grape stem weight, grape berries weight, weight of ten grape berries, grape berry length, grape berry width, seeds weight, grape berry flesh weight). To establish relationships among individual polyphenols, TPC, TAC, and RSA, correlation analysis was performed. According to Table 8, TAC had significant correlations with chlorogenic acid (r = 0.91, *p* < 0.00001), neo-chlorogenic acid (r = 0.98, *p* < 0.00001), rutin (r = 0.84, *p* < 0.0001), isorhamnetin 3-*O*-rutinoside (r = 0.88, *p* < 0.00001), and myricetin (r = 0.79, *p* < 0.001). Other significant correlations (*p* < 0.00001) were observed between glucosides of quercetin and kaempferol (r = 0.94), TPC and RSA (r = 0.94), quercetin and kaempferol (r = 0.94), chlorogenic and neo-chlorogenic acids, and rutin and isorhamnetin 3-*O*-rutinoside (both r = 0.90). Neo-chlorogenic acid was also positively correlated with rutin and isorhamnetin 3-*O*-rutinoside (*p* < 0.0001), with correlation factors of 0.84 and 0.86, respectively, while rutin was associated with quercetin 3-*O*-rhamnoside (r = 0.80, *p* < 0.001).

## 3. Discussion

### 3.1. Grape Cluster Characteristics

The obtained results indicate the specificity of the analyzed varieties compared to the results of previous research on the production characteristics of these varieties grown in the same region, as well as in the closer (Montenegro, Croatia, Serbia) and more distant regions.

The tested varieties in the Herzegovina region achieve yields that are above all a varietal characteristic and a reflection of the growing conditions. Higher yields per vine (Banjanin, 2022) [1] were recorded for the autochthonous varieties Blatina (4.7 kg/vine) and Vranac (4.6 kg/vine), and somewhat lower for the introduced varieties Merlot (3.9 kg/vine) and Cabernet Sauvignon (2.9 kg/vine). The Vranac variety in the surrounding area achieves similar yields [28], while certain deviations in the yield per vine have been recorded in the use of these varieties in the surrounding countries [29,30,31].

According to the results of our research and literary references, Cabernet Sauvignon is a variety with small grape clusters and small grape berries. The average values of the grape cluster weight obtained in our research at the Mostar locality (151.2 g) correspond to the upper limit of the average cluster weight (67 g–171 g), as stated by several authors [29,32,33,34,35].

The average grape cluster weight of the Merlot variety (and certain clones) ranges from 67 to 210 g [31,32,36,37] and it is significantly lower compared to the results of our research at both localities (226 g—Trebinje; 294 g—Mostar). During the research period, a significant variation in grape cluster weight and number of berries was observed in the Merlot variety at both localities. A similar tendency in the variability of grape cluster weight, grape berry weight, and number of grape berries was observed in the research of the mentioned variety at the Trebinje locality during previously conducted research in the period 2016–2017 [38]. In 2017, higher values of the mentioned parameters were recorded due to more favorable weather conditions (higher amount of precipitation in July, August, and September).

The observed grape cluster weight of the Vranac variety, grown in the agro-ecological conditions of the surrounding countries (Montenegro and Serbia), was lower compared to the results of our research (272.1 g–414.2 g) and ranged from 178 to 300 g [39,40,41], except for certain Vranac clones (Vranac clone 2 and Vranac clone 5) whose grape cluster weight was slightly higher (327 g and 336 g, respectively) [28]. The results of recently conducted research on the production characteristics of the Vranac variety grown in the Trebinje area (2016–2017 and 2016–2018) [1,42] are in accordance with the results of our research in terms of the average grape cluster weight (355 g and 350 g, respectively) at both localities, while the weight of 100 berries (314 g and 341 g, respectively) was slightly higher compared to our results, and the determined number of berries in a bunch (130–148) showed a deviation, but only in relation to the location of Mostar. The relatively high agreement of these results with ours indicates that the influence of the agro-ecological conditions of the locality on the examined physical characteristics was significant.

Although the Blatina variety had the highest recorded grape cluster weight during the research period (428.7 g), this variety has the greatest variability in terms of the mentioned characteristic, as well as the number of grape berries in a grape cluster. The results of multi-year studies conducted by other authors indicate the occurrence of variability in the ampelographic characteristics of the Blatina variety, grown in the region of the Herzegovina vineyards. The average weights of a grape cluster and 100 grape berries of the Blatina variety, according to [43], were 250 g and 204 g, respectively, and the average number of grape berries in a grape cluster was 103, which is lower compared to our results. According to [44], the average grape cluster weight of this variety (2008–2009) ranged from 125.00 g to 189.88 g, while average weight of 100 berries was 192.75 g.

The analysis of the basic ampelographic characteristics of the Blatina variety in the collection plantation at the Višići locality in Herzegovina [45] shows a significant agreement with the results of our research.

Climatic factors have a significant influence on the basic characteristics of the Blatina grape variety, whether it is a microlocality in the area of Herzegovina [46,47] or during cultivation in regions with different climatic characteristics [48,49]. The average grape cluster weight of the Blatina variety grown in the agro-ecological conditions of Serbia (1981–1998) was 273.00 g [48], while in the conditions of Macedonia, it was 200.00 g [49].

The average grape berry weight (0.64 g–1.6 g) and the number of seeds in the grape berry (1.08–2.03) in the variety Cabernet Sauvignon correspond to results that can be considered a varietal characteristic, bearing in mind the positive correlation with the results of other authors who dealt with this issue [8,50,51,52]. Similar results were recorded in the analysis of the number of grape berries in a grape cluster, which ranges from 65 to 164 [53,54,55,56], except for a slightly higher number of berries at the Trebinje locality during 2020. The grape berry weight of the Merlot variety is significantly influenced by production conditions, as evidenced by many literature references. The results obtained in our research are in accordance with the statements of the several authors [57,58,59,60,61].

Cultivation of the Merlot variety in different agroclimatic conditions significantly affects the specific characteristics of the bunch, which are characterized by a slightly smaller number of berries in the bunch [62,63] or their size [64].

According to [47], significant variation in the characteristics of the Blatina variety can be attributed to varietal specificities regarding the pollination and fertilization process, and thus the fruit set. According to the same author, the number of grape berries in a grape cluster ranged from 53 to 73, while the average grape berry weight varied from 2.88 to 4.28 g, and the number of seeds per grape berry varied from 1.23 to 1.81.

### 3.2. Grape Quality

The high quality of Cabernet Sauvignon grapes was also shown by the results of the must physicochemical analysis, which is also confirmed by the research of other authors [65,66,67,68]. The results of studies on grape quality of this variety in neighboring regions (Croatia, Serbia) [31,69,70] show that the total soluble solids (TSS) content was at the level of the lowest measured value of TSS in our research (Trebinje, 2021—22.8 °Brix), while the TA content was slightly higher (6.80 g·L^−1^–9.20 g·L^−1^). This quality of grapes can partly be justified by the influence of the colder climatic conditions of the mentioned regions. Higher pH values (Mostar, 3.65 and 3.70) are relatively in line with the statements of other authors [57,71]. The high values of TSS in the grape juice of the Merlot variety at both analyzed localities in the years of the research are relatively in line with the results of some other authors [63,72,73], while the level of determined titratable acids (TA) was mostly insignificantly higher compared to the results of the same authors. Higher values of TSS (24.0 °Brix and 24.4 °Brix) in Trebinje were observed during research on the grape quality of this variety in the earlier period (2016–2017) [38]. During these studies, a significant variability in terms of TA content was observed (8.25 g·L^−1^ and 5.7 g·L^−1^), which was also manifested in the case of our research at Mostar locality in 2020.

In contrast to the above, according to the results of multi-year research conducted on this variety and its individual clones [30,61] grown in the neighboring area, the TSS content in the must was considerably lower while the level of TA was higher compared to our results.

On the basis of a comparative analysis of the chemical composition of the Blatina varieties’ must with the available literature data, it can be said that the measured TSS values at Trebinje locality were most in line with the results published by [44], which ranged from 18.0 °Brix to 23.5 °Brix, while the level of TA, according to the same author, was in the range from 6 g·L^−1^ to 8 g·L^−1^, which is in accordance with the results of our research determined in samples from Mostar area. The lowest observed TA content in this variety is very close to the level noted by [1] (4.57 g·L^−1^). Similar results are reported by [46] (4.22 g·L^−1^), whose two-year research, in addition to the above, clearly shows the influence of different growing localities on the occurrence of variations in the TSS content of this variety grapes (17.6% Brix–20.9% Brix in 2008, and 17.1% Brix–20.2% Brix in 2009). According to the results of a two-year study on the Blatina variety in the area of Mostar [74], the TSS level in Blatina must range from 19.0 °Brix to 20.5 °Brix; the content of total titratable acids from 5.8 g·L^−1^ to 6.8 g·L^−1^; and the pH value was 2.9 and 3.3 in 2020 and 2021, respectively, whereby a significant influence of the harvest year, i.e., climatic factors on all analyzed parameters was observed. We find fairly similar results of the chemical composition of this variety also grown in Mostar (TSS—220 g·L^−1^, TA—6.05 g·L^−1^, and pH 3.39) in the research of [75].

The results of research on the chemical characteristics of the Vranac variety grapes [76], conducted in the primary growing region of this variety in Montenegro, showed the possibility of reaching high TSS values in the must, which is in line with the results of our research during 2020 at both localities. In the case of our research, high values of TSS were accompanied by a low content of TTA, in contrast to the results of research conducted by [77], where the average level of TTA was low established also at a significantly lower average TSS level. According to [78], the relatively low level of TTA recorded during their analysis (4.0 g·L^−1^ and 5.6 g·L^−1^) is typical for the largest cultivation region of the Vranac variety in Montenego (Podgorica district).

The results of the measured pH values in our research are generally comparable to the results of a number of authors [71,79,80].

### 3.3. Insight into the Phenolic Composition of the Investigated Grape Samples

The results obtained in this work once again confirmed that the content of individual polyphenols (phenolic profile) strongly depends on the grape variety [81,82]. Flavonols were the most abundant of all flavonoids in the examined grape extracts, likely originating mainly from grape berry skins [83]. This group of polyphenolic phytochemicals have been studied extensively because of their antioxidant potency and other biological activities [84]. Literature data indicate that the contents of flavonols varied among grape varieties and that they are found present mostly in the form of 3-*O*-glycosides [82], and the present study confirmed these statements.

Moreover, quercetin 3-*O*-glucoside was already documented as the predominant compound found present in most grape varieties. The present study confirmed all these statements. Among hydroxycinnamic acids, the contents of chlorogenic and neo-chlorogenic acids were notably higher when compared to the content of caffeic acid. These findings are significant as the previous literature [85] highlighted benefits of chlorogenic acids in controlling oxidative and inflammatory stress conditions.

Interestingly, the highest values of individual polyphenols were found mostly in samples from Trebinje, except for chlorogenic acid, isorhamnetin 3-*O*-rutinoside, myricetin, and eriodictyol, whose highest values were found in the samples from Mostar. As the production and biosynthesis of polyphenols significantly depends on climate [23], the obtained differences in phenolic contents might be attributed to environmental conditions.

Mostar belongs to a modified humid subtropical climate (cold, humid winters and hot, drier summer), while the climate in Trebinje is Mediterranean, with short mild winters and long hot summers (subtropical climate), typical for the southern Adriatic coastal areas [84]. On the other hand, no trends were observed in the contents of polyphenols depending on the grape harvest year.

According to the obtained results, none of the varieties stood out with particularly high or low values of TPC and RSA. A similar trend was observed by [86] when they examined TPC and RSA of the seeds of the same four grape varieties. TPC and RSA of the Merlot and Cabernet Franc mature grape berries, reported by [87], were in the ranges 2.76–10.89 g GAE/kg and 26.81–80.48 mmol TE/kg(respectively), which was in accordance with our results. The RSA value (34.51 µmol TE/g) obtained for Isabel grape sample (the hybrid of V. labrusca L. × V. vinifera L.) originated from Turkey [88], was in the line with our results, while TPC and TAC were somewhat higher (1101.61 mg/100 g and 341.88 mg mal 3-glu/100 g, respectively). As for TAC, Vranac grapes had the highest values when compared to the rest of the analyzed varieties. The grape skins of this indigenous variety were already proven to be a rich source of anthocyanins, with the TAC ranging from 3.3 g mal 3-glu/kg (Mlava vineyard area, Serbia) to 7.58 g mal 3-glu/kg (“Ćemovsko polje”, Montenegro) of frozen skin weight [89,90]. Additionally, when PCA was applied on the results obtained for individual polyphenols, TPC, TAC, and RSA, all grape samples belonging to the Vranac variety differed from other varieties on the basis of higher contents of some polyphenols (neo-chlorogenic acid, rutin, isorhamnetin 3-*O*-rutinoside, myricetin, and chlorogenic acid) and TAC. Those findings are important indicators of the quality of this Montenegrin indigenous grape variety that was already characterized by great diversity of phenolic compounds in all berry tissues—skin, seed, and pulp [90]. Polyphenols were mainly responsible for the grouping of the Vranac variety samples, but the indigenous variety from Bosnia and Hercegovina, Blatina, also stood out with higher contents of some polyphenols (dihydroquercetin, kaempferol 3-*O*-glucoside, isorhamnetin, and naringenin—sample from Trebinje (2020), and phlorizin and quercetin 3-*O*-rhamnoside—sample from Trebinje (2021)). When PCA was extended to all parameters, the two autochthonous Balkan varieties (Vranac—Montenegro and Blatina—Bosnia and Hercegovina) were separated from the remaining two international grapevine varieties (Cabernet Sauvignon and Merlot) due to higher contents of three polyphenols (quercetin 3-*O*-rhamnoside, and chlorogenic and neo-chlorogenic acids) and some physicochemical parameters.

In the search for significant correlations among polyphenols, TPC, TAC, and RSA, we encountered interesting results. According to Pearson’s correlation matrix, TAC had the largest number of significant correlations, with chlorogenic acid, neo-chlorogenic acid, rutin, isorhamnetin 3-*O*-rutinoside, and myricetin. These flavonoids could be considered as compounds that are in direct synergy with anthocyanins present in the extracts, originating from the red grape skins. In previous publications, myricetin was already described as a constituent characteristic for red skin grapes and wines [87,91]. Moreover, the authors of [91] reported higher contents of chlorogenic acid and rutin in the skins of red grapes when compared to the white ones. The satisfactory correlation coefficient obtained between TPC and RSA indicated that polyphenols are compounds that mainly contribute to antioxidant activity of the analyzed grape samples. All significant correlations obtained in the present work support the assertion that polyphenols have interactive and complementary effects. The results of [92] showed the potential pharmacological and therapeutic superiority of a combination of polyphenols with respect to their individual compounds in clinical medicine and human nutrition. The benefits of the whole extract (mix of polyphenols) were greater when compared to the extracts obtained by fractionation and isolation of polyphenols, which indicates a synergistic effect of phenolic compounds.

## 4. Materials and Methods

### 4.1. Plant Material

The research was carried out through a field experiment in production vineyard in Trebinje (vineyard “AD Popovo polje”, Dubljani) and Mostar (a vineyard owned by Fran Ostojić, Potpolje). The vineyards are in the region of Herzegovina and belong to the Mostar vineyards, according to the official regionalization of wine production in Bosnia and Herzegovina. The analysis included the old varieties Blatina and Vranac.

The Blatina variety is an indigenous variety in B&H, while Vranac is a Montenegrin indigenous variety, which is intensively cultivated in Herzegovina. Cabernet Sauvignon and Merlot varieties were used as comparative varieties in the research, which in recent years have been increasingly represented in the structure of varieties for red wines in B&H. All varieties were grafted on Kober 5BB rootstock. Both vineyards are in full productivity, considering that the vineyard in Trebinje was raised in 2013, and in vineyard in Mostar in 2010. The plant spacing for all analyzed varieties was 2.8 m × 1.0 m. The training system was Moser cordon two-armed cordon. The research was conducted in 2020 and 2021. Using the method of random sampling, 60 plants from each locality were selected for analysis so that each variety was represented by 15 plants per locality. Grapevines were grouped in 3 repetitions with 5 grapevines each. Mixed pruning was carried out in all the analyzed grapevines during the dormancy period, where 12 fertile buds were left per grapevine. Laboratory research was carried out in the horticulture laboratories of the Faculty of Agriculture, University of Banja Luka, and in the chemistry laboratory of the Innovative Center of the Faculty of Chemistry, University of Belgrade.

### 4.2. Climatic Conditions

The available average monthly temperatures and monthly precipitation sums for the analyzed localities were obtained from the synoptic stations Trebinje of the Republic of Srpska Hydrometeorological Institute and Mostar of the Federal Hydro-Meteorological Institute [93,94,95]. The geographic coordinates of the synoptic station in Trebinje are 42°43′ N, 18°21′ E, and it is located at an altitude of 276 m, and the synoptic station in Mostar is located at 43°21′ N, 17°48′ E, at an altitude of 99 m. With the help of the collected data, a basic climatological analysis of both localities was carried out for the needs of grapevine cultivation. To assess the impact of climate change, both analyses were performed for the twenty-year period from the past, namely, 2000–2019. The same analyses were performed for the years 2020 and 2021, in which the experiment was conducted. The following data were calculated as part of the basic climatological analysis: Tmin—average monthly, annual, and vegetation period (from 1 April to 31 October) minimum temperature values (°C); Tmax—average monthly, annual, and vegetation period (from 1 April to 31 October) maximum temperature values (°C); Ts—average monthly, annual, and vegetation period (from 1 April to 31 October) average temperature values (°C); RR—monthly, annual, and vegetation (from 1 April to 31 October) precipitation sums (mm); GDD—average sum of active temperatures, i.e., the sum of mean daily temperatures higher or equal to 10 °C during the vegetation period. The climate of the wine-growing localities in Trebinje and Mostar were based on the calculated Winkler index—WI or GDD [96].

### 4.3. Grape Cluster and Grape Juice Quality

The harvest was performed at the time of optimal maturity for the analyzed varieties and when the TSS (total soluble solids) content was between 20 and 25 °Brix (BBCH 89 phase). The number of grape clusters was determined on ten grapevines of each of the studied varieties. Individual clusters and mature grape berries of each variety were collected, counted, and weighed. Ten representative clusters from each treatment were selected. The number of grape berries per cluster was recorded at maturity. The weight of grapes in the cluster and the weight of an individual grape berry were measured with a digital scale (KERN 440, Balingen, Germany). Afterwards, the seeds from each mature grape berry were extracted and washed to remove pulp. The weight of the total number of seeds of 10 berries was determined by measuring on a digital scale. Mechanical analysis was performed on grape cluster at optimal maturity (10 grape clusters and 100 grape berries).

The basic parameters of grape juice quality were determined by analyzing the total soluble solids content—sugar (TSS, °Brix) in the juice, total titratable acids (TTA), and juice pH value. The sugar content was measured with a digital refractometer (Atago-Pal-3), while the total titrable acidity (g·L^−1^ tartaric acid) was determined by an acid neutralization method with 0.1 N NaOH solution. The pH value of grape juice was determined with a pH meter (Hanna HI2211).

### 4.4. Chemical Analysis

#### 4.4.1. Chemicals and Materials

Standards of polyphenols and 6-hydroxy-2,5,7,8-tetramethylchroman-2-carboxylic acid (Trolox) were purchased from Sigma-Aldrich (Steinheim, Germany); methanol, acetonitrile (both HPLC grade), formic acid, ethyl acetate, sodium carbonate, and Folin–Ciocalteu reagent were from Merck (Darmstadt, Germany); and 2, 2-diphenyl-1-picrylhydrazyl radical (DPPH·) was purchased from Fluka AG (Buch, Switzerland). Syringe filters (13 mm, PTFE membrane 0.45 µm) were obtained from Supelco (Bellefonte, PA, USA). All standard solutions and dilutions were prepared using ultrapure water (MicroPure water purification system, 0.055 µS/cm, TKA, Niederelbert, Germany).

#### 4.4.2. Extraction of Polyphenols from Grape Samples

The extraction procedure was performed according to [83]. Frozen grape berries (10 g) were first homogenized with pestle and mortar. Each grape sample was extracted with 50 mL of acidified MeOH (0.1% HCl, *v*/*v*) in an ultrasonic bath for 30 min (at room temperature, in the dark). The extracts were filtered using gauze (4 layers), and the clear filtrates supernatants were collected. The procedures were repeated two more times. All three fractions were combined and evaporated to dryness by rotary evaporation (IKA^®^-Werke GmbH and Co. KG, Staufen, Germany) under reduced pressure at 40 °C. The residues were then dissolved in MeOH/H_2_O (60:40, *v*/*v*) in a 50 mL volumetric flask. The prepared extracts were stored in the freezer until further analyses. Before spectrophotometric and LC–MS measurements, the extracts were filtered through 0.45 μm membrane filters.

#### 4.4.3. Determination of Total Phenolic Content (TPC), Radical Scavenging Activity (RSA), and Total Anthocyanin Content (TAC) of Grape Samples

The assessments of TPC, RSA, and TAC were performed in triplicate using spectrophotometric procedures described by [83]. All measurements were made on a Cintra 6 UV–VIS spectrophotometer (GBC Scientific Equipment Ltd., Hampshire, IL, USA). The Folin–Ciocalteu method was used for the determination of TPC. According to this method, the reaction mixtures are prepared by combining the extracts, Folin–Ciocalteu reagent (10%), sodium carbonate solution (7.5%), and water. After incubation for two hours (at room temperature, in the dark), the absorbance was measured at 765 nm. According to standard curve generated with different concentrations of gallic acid, TPC values were expressed as gram of gallic acid equivalent per kilogram of frozen berries weight (g GAE/kg). RSA was determined using the DPPH˙ method, which includes mixing of the obtained phenolic extracts and DPPH radical methanol solution, incubation during 1 h, and absorbance monitoring at 515 nm. Trolox was used as a standard, and a calibration curve was displayed as a function of the percentage of inhibition of DPPH˙. The results for RSA were expressed as milimoles of Trolox equivalents per kilogram of frozen sample weight (mmol TE/kg). TAC was assessed by the pH-differential method by diluting extracts with buffers pH 1.0 (KCl, 0.025 mol/L) and pH 4.5 (NaOAc/HOAc, 0.4 mol/L) and measuring the absorbance at 510 and 700 nm. TAC was expressed as gram of malvidin-3-glucoside equivalents per kilogram of frozen grape weight (mal-3-glu/kg).

#### 4.4.4. Estimation of Phenolic Profile (Characterization of Individual Polyphenols)

Determination of the phenolic profile in the analyzed grape samples was performed on a Dionex Ultimate 3000 ultra-high-performance liquid chromatograph (UHPLC, Thermo Fisher Scientific, Bremen, Germany) with an ultraviolet multi-diode detector (DAD) and connected to a triple quadrupole weight spectrometer (TSQ Quantum Access MAX, Thermo Fisher Scientific, Basel, Switzerland). Separation was achieved on a Syncronis C18 column (100 × 2.1 mm, 1.7 lm particle size) at 40 °C. All experimental conditions were previously described by [83].

Detection of polyphenols was acquired in the negative mode on a TSQ Quantum Access Max triple-quadrupole weight spectrometer (Thermo Fisher Scientific, Basel, Switzerland) with a heated electrospray ionization (HESI) source. The parameters and ion source settings were the same as in [83]. For instrument control, Xcalibur software 2.2 (Thermo Fisher, Bremen, Germany) was used, while phenolic compounds were identified by direct comparison with commercial standards. The polyphenols content was expressed as mg/kg.

### 4.5. Statistical Analysis

The statistical analysis of grape cluster and grape juice quality were performed using Statgraphics Centurion. The obtained results were subjected to analysis of variance (ANOVA) according to a factorial design, where the sources of variation were year and variety, as well as their interaction. Comparison of means was performed by the Tukey test (α = 0.05). The results are presented as the mean value ± standard error of mean (SEM).

PLSTool Box software package for MATLAB (Version 7.12.0) was used for principal component analysis (PCA) for chemical components of grape analysis. Before the PCA, all data were group-scaled, and the singular value decomposition algorithm (SVD) and a 0.95 confidence level for Q and Hotelling T2 limits for outliers were chosen. The Number Cruncher Statistical System (NCSS) software package (www.ncss.com, accessed on 4 October 2022) was used to calculate Pearson’s correlation coefficients. Although a *p* ≤ 0.05 was generally used, we chose to use 0.001, 0.0001, and 0.00001 in order to indicate the greater significance of the differences.

## 5. Conclusions

The largest deviations of cluster weight and number of berries in the research period, in both localities, were observed in the Merlot variety. The variety Cabernet Sauvignon showed the highest stability of the observed characteristics. Intervarietal differences were also manifested at the berry level, i.e., in berry mass and number of berry seeds. Larger deviations in the mass of berries in the years of research were found in the Blatina (Trebinje) and Merlot (Mostar) varieties, while the deviation in the number of seeds per berry was the most noticeable in the Blatina variety. The quality of the grapes was generally a reflection of the weather conditions during the research, i.e., temperatures, which in principle favored the accumulation of a higher amount of sugar (TSS) in most varieties, while the lower TSS values found in grapes of the Blatina variety can also be attributed to varietal specificity. On the other hand, the drop in acid levels (TTA) in certain varieties (Blatina, Vranac, Merlot) may also reflect extremely high temperatures during the ripening period, the highest average values of which reached values of 37.6 °C (Trebinje) and 40.8 °C (Mostar). This research once again confirmed the strong influence of grape variety on the berries phenolic profile. The most abundant polyphenols in analyzed grape samples were quercetin 3-*O*-glucoside and catechin gallate, followed by kaempferol 3-*O*-glucoside, rutin, and isorhamnetin 3-*O*-rutinoside. The highest values of individual polyphenols were found mostly in samples from Trebinje, while no trends were observed when harvest years were considered. The Vranac grape variety differed from other varieties in terms of its higher contents of neo-chlorogenic acid, rutin, isorhamnetin 3-*O*-rutinoside, myricetin, chlorogenic acid, and TAC, while Blatina grape samples stood out with higher contents of dihydroquercetin, kaempferol 3-*O*-glucoside, isorhamnetin, naringenin, phlorizin, and quercetin 3-*O*-rhamnoside. Correlation analysis revealed some strong associations, pointing to interactive and complementary effects among polyphenols. To the best of our knowledge, this is the first characterization of the phenolic profile of indigenous grape variety Blatina. Cultivation of autochthonous varieties in Herzegovina may have the potential to make viticulture climate smart, genetically diverse, and sustainable, bearing in mind the significant climate changes that have occurred in recent years. At the same time, these varieties provide wines with particular characteristics for this production region, which has multiple economic significance. Producers must keep these facts in mind when they decide to introduce international varieties into their production and define their percentage representation in relation to autochthonous varieties.

## Figures and Tables

**Table 1:** Basic climatic conditions at the Trebinje locality during 2020–2021 and average data for the period of 2010–2019.

	Mean Temperature			Av. Max. T. * (°C)		Av. Min. T. (°C)		GDD (°C)		Rain (mm)		
	Aug.–Sep.	Apr.–Oct.	Year	Aug.–Sep.	Apr.–Oct.	Aug.–Sep.	Apr.–Oct.	Aug.–Sep.	Apr.–Oct.	Aug.–Sep.	Apr.–Oct.	Year
2020	23.6	20.0	14.4	30.6	26.1	18.8	15.0	829.6	2140.0	189.4	543.4	1124.4
2021	22.8	19.6	14.9	29.6	26.0	18.0	14.8	783.9	2054.4	121.8	496.9	1691.8
00–19	23.3	20.8	15.7	29.1	26.1	17.4	15.1	747.9	2372.7	194.2	705.3	1683.7

(* Av. Max. T.—average maximum temperature; Av. Min. T.—average minimum temperature; GDD—growing degree days).

**Table 2:** Basic climatic conditions at the Mostar locality during 2020–2021 and average data for the period 2010–2019.

	Mean Temperature			Av. Max. T. * (°C)		Av. Min. T. (°C)		GDD (°C)		Rain (mm)		
	Aug.–Sep.	Apr.–Oct.	Year	Aug.–Sep.	Apr.–Oct.	Aug.–Sep.	Apr.–Oct.	Aug.–Sep.	Apr.–Oct.	Aug.–Sep.	Apr.–Oct.	Year
2020	25.0	21.1	16.3	31.8	27.4	19.5	15.5	912.0	2375.4	196.9	521.1	1066.7
2021	24.4	21.1	16.0	31.8	27.8	18.6	15.5	875.4	2375.4	136.4	454.0	1541.5
00–19	24.3	21.4	16.1	30.5	27.4	18.2	15.1	875.1	2555.3	191.5	685.8	1491.6

(* Av. Max. T.—average maximum temperature; Av. Min. T.—average minimum temperature; GDD—growing degree days).

**Table 3:** Grape cluster characteristics of analyzed varieties during the research period at the Trebinje locality.

	Grape Cluster Weight (g)	Number of Grape Berries in Grape Cluster	Weight of Ten Grape Berries	Number of Seeds in Ten Grape Berries
Cabernet Sauvignon/2020	198.4 ± 9.4 ^c^	207.9 ± 11.8 ^a^	11.4 ± 0.6 ^d^	16.5 ± 1.3 ^b^
Merlot/2020	278.4 ± 10.8 ^b^	206.4 ± 10.1 ^a^	15.2 ± 0.6 ^c^	20.4 ± 1.1 ^ab^
Blatina/2020	373.9 ± 23.5 ^a^	194.9 ± 21.7 ^ab^	22.3 ± 1.4 ^b^	17.5 ± 1.1 ^ab^
Vranac/2020	339.9 ± 20.9 ^a^	139.0 ± 15.9 ^bc^	29.8 ± 0.7 ^a^	22.7 ± 1.5 ^a^
Cabernet Sauvignon/2021	192.3 ± 12.9 ^c^	155.2 ± 12.2 ^abc^	12.8 ± 0.4 ^cd^	17.1 ± 1.0 ^ab^
Merlot/2021	173.9 ± 16.2 ^c^	121.0 ± 13.4 ^c^	15.1 ± 0.7 ^c^	19.2 ± 1.0 ^ab^
Blatina/2021	325.3 ± 38.6 ^a^	115.2 ± 14.4 ^c^	29.8 ± 0.9 ^a^	22.8 ± 1.8 ^a^
Vranac/2021	272.1 ± 28.8 ^a^	130.1 ± 19.0 ^bc^	26.6 ± 0.6 ^a^	18.5 ± 1.5 ^ab^
Year (Y)	16.13 ***	33.21 ***	6.60 ***	0.02 ^ns^
Variety (V)	25.26 ***	3.94 *	205.47 ***	3.26 *
Y × V	2.10 ^ns^	3.15 *	16.64 ***	4.31 **
Mean Values (± Standard Error of Mean)				
Year				
2020	297.7 ± 13.5 ^a^	187.1 ± 8.7 ^a^	19.6 ± 1.2 ^b^	19.3 ± 0.7 ^a^
2021	240.9 ± 15.9 ^b^	130.4 ± 7.6 ^b^	21.1 ± 1.2 ^a^	19.4 ± 0.7 ^a^
Variety				
Cabernet Sauvignon	195.4 ± 7.8 ^c^	181.6 ± 10.2 ^a^	12.1 ± 0.4 ^d^	16.8 ± 0.8 ^b^
Merlot	226.1 ± 15.3 ^c^	163.7 ± 12.8 ^ab^	15.1 ± 0.4 ^c^	19.8 ± 0.8 ^a^
Blatina	349.6 ± 22.6^a^	155.1 ± 15.6 ^ab^	26.0 ± 1.2 ^b^	20.2 ± 1.2 ^a^
Vranac	306.0 ± 19.0 ^b^	134.5 ± 12.1 ^b^	28.2 ± 0.6 ^a^	20.6 ± 1.1 ^a^

^a–d^ Different letters within the same column indicate statistically significant difference at *p* < 0.05 by Duncan’s test; ***, **, * significant at *p* < 0.001, *p* < 0.01 and *p* < 0.05, respectively; ns—not significant.

**Table 4:** Grape cluster characteristics of the analyzed varieties during the research period at the Mostar locality.

	Grape Cluster Weight (g)	Number of Grape Berries in Grape Cluster	Weight of Ten Grape Berries	Number of Seeds in Ten Grape Berries
Cabernet Sauvignon/2020	152.9 ± 10.7 ^cd^	120.7 ± 8.0 ^bc^	12.6 ± 0.3 ^d^	15.5 ± 0.4 ^d^
Merlot/2020	325.9 ± 37.8 ^ab^	195.1 ± 21.8 ^a^	20.1 ± 0.7 ^c^	21.6 ± 1.1 ^abc^
Blatina/2020	229.1 ± 22.3 ^bcd^	78.9 ± 7.8 ^c^	32.1 ± 1.1 ^ab^	25.7 ± 1.3 ^a^
Vranac/2020	379.2 ± 28.8 ^a^	156.2 ± 15.4 ^abc^	28.1 ± 1.0 ^b^	18.3 ± 1.1b ^cd^
Cabernet Sauvignon/2021	149.5 ± 11.2 ^d^	125.7 ± 8.4 ^abc^	12.6 ± 0.3 ^d^	16.0 ± 0.9 ^d^
Merlot/2021	261.6 ± 11.3 ^bc^	184.4 ± 18.3 ^ab^	15.9 ± 0.4 ^d^	18.1 ± 0.8 ^cd^
Blatina/2021	428.7 ± 27.5 ^a^	145.0 ± 14.2 ^abc^	33.0 ± 1.3 ^a^	18.7 ± 0.8b ^cd^
Vranac/2021	414.2 ± 31.7 ^a^	161.1 ± 8.3 ^abc^	29.1 ± 1.0 ^b^	22.5 ± 1.0 ^ab^
Year (Y)	9.59 **	4.65 *	1.08 ^ns^	4.50 *
Variety (V)	58.97 ***	21.83 ***	223.12 ***	15.88 ***
Y × V	17.55 ***	5.04 **	3.76 *	12.61 ***
Mean Values (± Standard Error of Mean)				
Year				
2020	271.7 ± 19.0 ^b^	137.7 ± 9.8 ^b^	23.3 ± 1.3 ^ns^	20.3 ± 0.7 ^a9^
2021	313.5 ± 21.3 ^a^	154.1 ± 7.1 ^a^	22.6 ± 1.4 ^ns^	18.8 ± 0.6 ^b^
Variety				
Cabernet Sauvignon	151.2 ± 7.6 ^c^	123.2 ± 5.7 ^c^	12.6 ± 0.2 ^d^	15.8 ± 0.5 ^c^
Merlot	293.8 ± 20.6 ^b^	189.8 ± 13.9 ^a^	18.0 ± 0.6 ^c^	19.9 ± 0.8 ^b^
Blatina	328.8 ± 28.7 ^b^	112.0 ± 10.9 ^c^	32.5 ± 0.8 ^a^	22.2 ± 1.1 ^a^
Vranac	396.7 ± 21.2 ^a^	158.7 ± 8.6 ^b^	28.7 ± 0.7 ^b^	20.4 ± 0.9 ^ab^

^a–d^ Different letters within the same column indicate statistically significant difference at *p* < 0.05 by Duncan’s test; ***, **, * significant at *p* < 0.001, *p* < 0.01 and *p* < 0.05, respectively; ns—not significant.

**Table 5:** Grape quality of the analyzed varieties during the research period at Trebinje locality.

	Total Soluble Solids (°Brix)	Titratable Acids (g/L)	pH
Cabernet Sauvignon/2020	24.3 ± 0.6 ^ab^	5.38 ± 0.02 ^d^	2.80 ± 0.02 ^d^
Merlot/2020	24.8 ± 0.6 ^a^	6.08 ± 0.01 ^c^	3.04 ± 0.01 ^bc^
Blatina/2020	23.5 ± 0.7 ^abc^	4.81 ± 0.02 ^e^	2.99 ± 0.02 ^bc^
Vranac/2020	23.4 ± 0.5 ^abc^	4.10 ± 0.01 ^f^	3.20 ± 0.01 ^ab^
Cabernet Sauvignon/2021	22.8 ± 0.4 ^abc^	6.78 ± 0.01 ^ab^	3.33 ± 0.01 ^a^
Merlot/2021	23.1 ± 0.2 ^abc^	6.47 ± 0.02 ^b^	3.12 ± 0.01 ^b^
Blatina/2021	21.5 ± 0.4 ^c^	7.08 ± 0.02 ^a^	2.89 ± 0.01 ^d^
Vranac/2021	22.4 ± 0.6 ^bc^	5.62 ± 0.06 ^d^	3.21 ± 0.04 ^ab^
Year (Y)	19.08 ***	5413.70 ***	103.87 ***
Variety (V)	3.14 *	1216.73 ***	42.31 ***
Y × V	0.37 ^ns^	405.59 ***	82.23 ***
Mean Values (± Standard Error of Mean)			
Year			
2020	24.1 ± 0.3 ^a^	5.10 ± 0.15 ^b^	3.04 ± 0.03 ^b^
2021	22.5 ± 0.2 ^b^	6.48 ± 0.12 ^a^	3.19 ± 0.03 ^a^
Variety			
Cabernet Sauvignon	23.6 ± 0.4 ^ab^	6.12 ± 0.21 ^b^	3.10 ± 0.08 ^b^
Merlot	23.9 ± 0.4 ^a^	6.28 ± 0.06 ^a^	3.08 ± 0.02 ^b^
Blatina	22.5 ± 0.5 ^b^	6.03 ± 0.34 ^c^	3.00 ± 0.01
Vranac	22.9 ± 0.4 ^ab^	4.80 ± 0.22 ^d^	3.20 ± 0.02 ^a^

^a–d^ Different letters within the same column indicate statistically significant difference at *p* < 0.05 by Duncan’s test; ***, * significant at *p* < 0.001, *p* < 0.01 and *p* < 0.05, respectively; ns—not significant.

**Table 6:** Grape quality of the analyzed varieties during the research period at the Mostar locality.

	Total Soluble Solids (°Brix)	Titratable Acids (g/L)	pH
Cabernet Sauvignon/2020	25.5 ± 0.23 ^ab^	5.10 ± 0.05 ^d^	3.65 ± 0.03 ^a^
Merlot/2020	26.3 ± 0.51 ^a^	4.68 ± 0.07 ^e^	3.50 ± 0.0 ^ab^
Blatina/2020	19.1 ± 0.29 ^d^	6.43 ± 0.01 ^b^	2.92 ± 0.01 ^c^
Vranac/2020	25.7 ± 0.47 ^ab^	4.51 ± 0.02 ^e^	3.20 ± 0.03 ^b^
Cabernet Sauvignon/2021	24.5 ± 0.28 ^b^	5.02 ± 0.01 ^d^	3.70 ± 0.02 ^a^
Merlot/2021	25.9 ± 0.43 ^ab^	5.96 ± 0.02 ^c^	3.34 ± 0.01 ^bc^
Blatina/2021	16.6 ± 0.23 ^e^	8.21 ± 0.01 ^a^	3.28 ± 0.01 ^bc^
Vranac/2021	21.9 ± 0.81 ^c^	5.83 ± 0.01 ^c^	3.55 ± 0.01 ^ab^
Year (Y)	39.59 ***	1890.91 ***	118.09 ***
Variety (V)	148.00 ***	1889.10 ***	310.66 ***
Y × V	6.33 ***	261.19 ***	89.14 ***
Mean Values (± Standard Error of Mean)			
Year			
2020	24.2 ± 0.64 ^a^	5.18 ± 0.16 ^b^	3.32 ± 0.06 ^b^
2021	22.2 ± 0.78 ^b^	6.26 ± 0.24 ^a^	3.47 ± 0.04 ^a^
Variety			
Cabernet Sauvignon	24.9 ± 0.23 ^b^	5.06 ± 0.03	3.68 ± 0.02 ^a^
Merlot	26.1 ± 0.32 ^a^	5.32 ± 0.19 ^b^	3.42 ± 0.03 ^b^
Blatina	17.8 ± 0.41 ^d^	7.32 ± 0.27 ^a^	3.09 ± 0.05 ^d^
Vranac	23.8 ± 0.73 ^c^	5.17 ± 0.19 ^c^	3.37 ± 0.05 ^c^

^a–d^ Different letters within the same column indicate statistically significant difference at *p* < 0.05 by Duncan’s test; *** significant at *p* < 0.001, *p* < 0.01 and *p* < 0.05, respectively; ns—not significant.

**Table 7:** Quantitative data on individual polyphenols, total phenolic content (TPC), radical scavenging activity (RSA), and total anthocyanin content (TAC) of frozen grape samples.

Parameter/Sample		Blatina				Vranac				Cabernet Sauvignon				Merlot			
		T2020	T2021	M2020	M2021	M2020	M2021	T2020	T2021	T2020	T2021	M2020	M2021	T2020	T2021	M2020	M2021
Hydroxycinnamic acids (mg/kg)																	
1	Chlorogenic acid	2.12	2.27	1.27	0.81	2.06	2.91	1.92	2.81	1.09	0.75	1.87	1.60	0.94	1.25	0.95	1.03
2	Neo-chlorogenic acid	2.14	2.03	0.95	1.38	2.21	2.21	2.20	2.59	1.16	1.14	1.56	1.38	1.18	1.29	1.17	1.28
3	Caffeic acid	0.20	0.11	0.07	0.14	0.08	0.07	0.15	0.11	0.10	-	0.10	0.15	0.08	0.12	0.10	0.06
Flavan-3-ols (mg/kg)																	
4	Catechin_gallate	8.83	17.46	15.72	7.72	13.91	18.15	17.99	25.02	8.53	14.90	11.06	9.00	20.51	15.60	15.15	18.65
Flavonols (mg/kg)																	
5	Rutin	4.65	8.48	4.02	5.99	10.63	8.95	10.67	13.39	3.68	4.71	2.28	2.38	4.07	5.21	3.54	3.79
6	Isorhamnetin 3-*O*-rutinoside	8.58	5.54	4.30	5.89	14.19	11.16	13.11	13.54	2.90	2.88	2.94	3.23	5.10	5.50	4.76	4.84
7	Quercetin	0.76	0.92	1.10	0.98	0.63	0.49	0.37	0.48	0.70	1.01	0.69	0.72	0.45	2.89	1.79	0.23
8	Dihydroquercetin	0.29	0.14	0.10	0.14	0.10	0.17	0.05	0.05	0.14	0.03	0.20	0.09	0.02	0.05	0.06	0.02
9	Quercetin 3-*O*-glucoside	30.79	25.99	18.50	9.03	14.74	12.64	10.29	12.05	13.53	37.56	18.45	24.17	17.69	20.76	33.46	3.84
10	Quercetin 3-*O*-rhamnoside	1.57	6.27	1.62	3.10	4.65	3.11	2.84	3.88	0.65	1.01	0.23	0.17	0.25	0.52	0.58	0.20
11	Kaempferol	0.93	0.72	0.66	0.61	0.69	0.35	0.60	0.57	0.54	0.66	0.55	0.55	0.54	1.52	1.28	0.34
12	Dihydrokaempferol	0.19	0.17	0.16	0.13	0.19	0.16	0.17	0.21	0.26	0.21	0.15	0.17	0.15	0.14	0.13	0.13
13	Kaempferol 3-*O*-glucoside	14.08	10.80	8.34	5.15	5.37	3.94	3.95	5.22	4.04	13.87	5.77	8.76	5.56	6.34	10.73	0.91
14	Myricetin	0.70	4.51	1.00	3.33	8.38	13.15	7.63	9.51	4.02	2.64	4.28	4.00	0.91	5.35	1.85	1.46
15	Isorhamnetin	1.18	0.25	0.52	0.23	0.36	0.28	0.30	0.13	0.41	0.44	0.43	0.63	0.29	0.88	0.68	0.16
Flavanones (mg/kg)																	
16	Naringenin	0.10	0.09	0.07	0.05	0.06	0.06	0.05	0.06	0.06	0.06	0.05	0.04	0.05	0.07	0.05	0.05
17	Eriodictyol	0.07	0.07	0.07	0.06	0.06	0.05	0.06	0.09	0.09	0.06	0.10	0.06	0.07	0.08	0.09	0.05
19	TPC (g GAE/kg)	5.70	6.40	4.59	3.17	4.83	6.43	5.58	6.30	4.42	6.76	6.11	5.25	4.04	4.17	3.82	4.20
20	RSA (mmol TE/kg)	35.57	41.69	33.59	27.58	33.80	43.81	39.85	43.49	34.55	48.55	45.09	38.34	29.42	32.24	32.37	35.40
21	TAC (g mal 3-glu/kg)	1.90	1.64	0.67	0.92	2.21	2.33	2.25	2.89	1.07	0.71	1.38	1.19	0.82	1.04	0.68	0.99

“-“ stands for not found; abbreviations: T for Trebinje and M for Mostar.

**Table 8:** Correlation analysis applied on the contents of individual polyphenols, TPC, RSA, and TAC in the analyzed grape samples *^a^*.

	1	2	3	4	5	6	7	8	9	10	11	12	13	14	15	16	17	18	19	20	21
1	1.00																				
2	0.90 ***	1.00																			
3	0.26	0.35	1.00																		
4	0.33	0.33	−0.38	1.00																	
5	0.68	0.84 **	0.05	0.56	1.00																
6	0.70	0.86 **	0.21	0.44	0.90 ***	1.00															
7	−0.32	−0.37	0.06	−0.14	−0.24	−0.29	1.00														
8	0.40	0.31	0.50	−0.58	−0.12	0.02	−0.11	1.00													
9	−0.17	−0.21	−0.05	−0.22	−0.33	−0.37	0.42	0.15	1.00												
10	0.61	0.70	0.07	0.27	0.80 *	0.62	−0.20	0.18	−0.14	1.00											
11	−0.22	−0.18	0.26	−0.11	−0.14	−0.11	0.91 ***	−0.02	0.52	−0.14	1.00										
12	0.22	0.22	−0.10	−0.10	0.23	0.12	−0.29	0.18	0.13	0.17	−0.24	1.00									
13	−0.08	−0.09	0.08	−0.26	−0.23	−0.27	0.31	0.29	0.94 ***	0.02	0.44	0.18	1.00								
14	0.75	0.71	−0.03	0.34	0.75	0.71	−0.16	0.02	−0.37	0.53	−0.22	0.18	−0.40	1.00							
15	−0.11	−0.16	0.47	−0.48	−0.43	−0.21	0.56	0.43	0.61	−0.38	0.69	−0.01	0.62	−0.37	1.00						
16	0.29	0.30	0.33	−0.05	0.15	0.13	0.18	0.54	0.33	0.39	0.32	0.20	0.53	−0.18	0.48	1.00					
17	0.03	−0.06	0.14	−0.05	−0.17	−0.24	0.27	0.16	0.32	−0.19	0.36	0.25	0.19	−0.11	0.20	−0.01	1.00				
18	0.32	0.25	−0.10	0.14	0.24	−0.05	−0.02	0.28	0.36	0.65	−0.04	0.14	0.50	0.02	−0.12	0.61	0.00	1.00			
19	0.66	0.56	−0.15	0.26	0.37	0.25	−0.31	0.24	0.29	0.35	−0.28	0.44	0.33	0.44	−0.07	0.24	0.08	0.52	1.00		
20	0.50	0.39	−0.31	0.25	0.25	0.10	−0.28	0.08	0.24	0.18	−0.32	0.37	0.21	0.42	−0.20	−0.01	0.12	0.41	0.94 ***	1.00	
21	0.91 ***	0.98 ***	0.30	0.36	0.84 **	0.88 ***	−0.40	0.24	−0.31	0.62	−0.24	0.30	−0.21	0.79 *	−0.20	0.19	−0.03	0.13	0.55	0.41	1.00

*^a^* Number of compounds corresponds to Table 1; *p* values: * *p* < 0.001, ** *p* < 0.0001, *** *p* < 0.00001.

## Data Availability

Not applicable.

## Supplementary Materials

The following supporting information can be downloaded at https://www.mdpi.com/article/10.3390/plants12040695/s1, Table S1: Physico-chemical parameters of investigated grape samples included in the principal component analysis (PCA). Figure S1: UV chromatograms of the analyzed grape samples recorded at 254 nm.
