# Supplementary material for: Exploration and Comparison of the Behavior of Some Indigenous and International Varieties (Vitis vinifera L.) Grown in Climatic Conditions of Herzegovina: The Influence of Variety and Vintage on Physico-Chemical Characteristics of Grapes
Source: Plants (Basel). 2023 Feb 4;12(4):695. doi: 10.3390/plants12040695 (PMC9961569; doi:10.3390/plants12040695)
Supplement: Supplementary file 1 [file plants-12-00695-s001.zip › Table S1.pdf]

Table S1. Physico-chemical parameters of investigated grape samples included in the principal component analysis (PCA).

parameter/sample		Blatina				Vranac				C.Sauvignon				Merlot			
		T2020	T2021	M2020	M2021	M2020	M2021	T2020	T2021	T2020	T2021	M2020	M2021	T2020	T2021	M2020	M2021
22	Cluster weight	400.33	325.29	229.01	463.97	379.22	414.17	379.28	272.14	198.39	192.33	152.93	149.53	278.40	173.87	325.91	283.52
23	Cluster length	15.72	16.44	15.02	17.00	17.30	19.96	18.77	15.97	16.51	16.31	14.00	14.20	18.76	15.46	20.20	19.50
24	Cluster width	11.64	12.14	10.25	13.64	9.80	10.95	10.16	11.63	10.06	10.39	8.85	8.90	11.40	9.67	10.51	11.35
25	Rachis weight	6.54	7.02	7.56	7.21	7.07	7.52	7.09	7.48	5.48	6.08	4.07	3.98	4.71	2.75	4.50	4.47
26	Stalk length	2.88	3.38	3.22	2.99	2.64	1.22	2.06	2.36	2.25	1.56	2.13	2.08	2.86	3.21	3.03	1.54
27	Nb. of berries	194.90	115.20	78.90	145.00	156.20	161.10	139.00	130.08	207.90	155.20	120.70	125.70	206.40	121.00	195.10	184.40
28	Berries weight	393.80	318.27	221.45	456.77	372.15	406.65	372.19	264.66	192.91	186.25	148.86	145.55	273.69	171.12	321.41	279.05
29	% of rachis	1.64	2.04	3.29	1.56	1.83	1.80	1.82	2.73	2.78	3.16	2.64	2.66	1.68	1.55	1.33	1.54
30	% of berries	98.36	97.96	96.71	98.44	98.17	98.20	98.18	97.27	97.22	96.84	97.36	97.34	98.32	98.45	98.67	98.46
31	Weight of 10 berries	22.26	29.79	32.00	33.00	28.41	29.04	29.76	26.56	11.39	12.84	12.63	12.62	15.18	15.09	20.06	15.87
32	Berry length	14.93	16.74	17.53	16.82	17.46	17.94	17.26	17.00	11.16	12.67	12.78	12.79	13.15	12.88	14.25	13.39
33	Berry width	14.72	16.85	16.91	16.83	15.41	16.36	16.08	15.51	11.40	12.41	12.49	12.41	13.18	12.82	14.16	13.19
34	Ratio L/W	1.01	0.99	1.04	1.00	1.13	1.10	1.07	1.10	0.98	1.02	1.02	1.03	1.00	1.00	1.01	1.01
35	Skin weight	2.45	1.20	1.78	1.42	3.03	1.82	2.04	1.63	0.87	0.90	1.20	1.19	1.03	0.74	2.71	1.11
36	Seeds weight	0.46	1.09	1.07	1.02	0.95	1.11	1.02	0.95	0.46	0.64	0.52	0.57	0.59	0.71	0.80	0.64
37	Nb. of seeds	17.50	22.80	25.70	18.70	18.30	22.50	22.70	18.52	16.50	17.10	15.50	16.00	20.40	19.20	21.60	18.10
38	Flesh weight	19.35	27.50	29.16	30.57	24.44	26.10	26.70	23.98	10.07	11.29	10.91	10.86	13.56	13.63	16.55	14.12
39	% Skin	11.39	4.02	5.62	4.35	10.61	6.33	6.89	6.13	7.56	7.02	9.51	9.51	6.82	5.02	13.38	7.08
40	% Seeds	2.01	3.62	3.31	3.13	3.32	3.86	3.43	3.59	3.98	5.03	4.10	4.47	3.89	4.71	3.97	4.07
41	% Flesh	86.60	92.35	91.07	92.52	86.07	89.81	89.67	90.28	88.46	87.95	86.39	86.02	89.29	90.27	82.65	88.85
42	TTS %	23.55	21.50	19.07	16.60	25.73	21.93	23.43	22.42	24.33	22.83	25.48	24.50	24.85	23.07	26.33	25.93
43	TTA gL^{-1}	4.88	7.13	6.44	8.21	4.51	5.82	4.10	5.56	5.37	6.77	5.10	5.02	6.07	6.48	4.68	5.96
44	pH	3.04	2.97	2.92	3.28	3.20	3.55	3.21	3.24	2.79	3.32	3.65	3.70	3.01	3.11	3.50	3.34
