# Supplementary figures and images for: Exploration and Comparison of the Behavior of Some Indigenous and International Varieties (Vitis vinifera L.) Grown in Climatic Conditions of Herzegovina: The Influence of Variety and Vintage on Physico-Chemical Characteristics of Grapes
Source: Plants (Basel). 2023 Feb 4;12(4):695. doi: 10.3390/plants12040695 (PMC9961569; doi:10.3390/plants12040695)

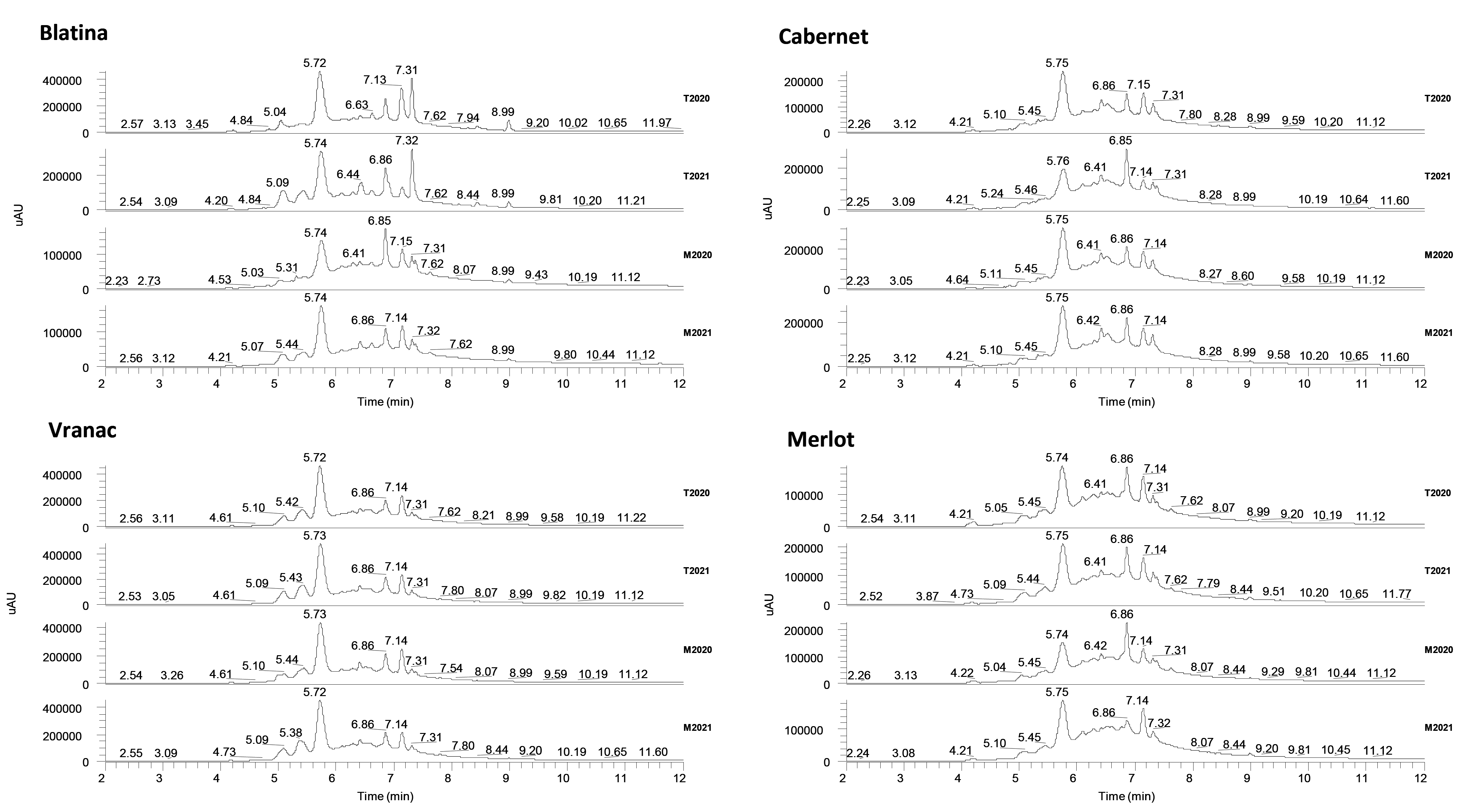

Supplement: Supplementary file 1 [file plants-12-00695-s001.zip › Figure S1.tif]
